# Implementation of the Age-Friendly Health System Framework at Four Clinical Sites: the UTHealth Houston Case

**DOI:** 10.1093/geroni/igaf122.3609

**Published:** 2025-12-31

**Authors:** Rafael Samper-Ternent, Mei Robson, Maureen Beck, Cindy Woolverton, Min Ji Kwak, Ezenwa Onyema, Cristina Murdock

**Affiliations:** University of Texas Health Science Center at Houston, Houston, Texas, United States; UTHealth Houston, Houston, Texas, United States; UTHealth Houston, Houston, Texas, United States; UTHealth Houston, Houston, Texas, United States; UTHealth Houston, Houston, Texas, United States; UTHealth Houston, Houston, Texas, United States; UTHealth Houston, Houston, Texas, United States

## Abstract

The Age-Friendly Health Systems (AFHS) framework promotes evidence-based care for older adults through the 4Ms: What Matters, Medication, Mentation, and Mobility. UTHealth Houston joined the Institute for Healthcare Improvement (IHI) System-Wide Spread Collaborative to expand AFHS adoption across four clinical sites: the Center for Healthy Aging (CHA), John S. Dunn Behavioral Health Sciences Center (Dunn), Harris Health System (HHS), and Memorial Hermann Hospital (MHH). We evaluate AFHS implementation using chart reviews from April 2024 to April 2025 to capture patient counts, 4Ms assessments, and corresponding clinical actions. We identify barriers to implementation via review of meeting notes and reports submitted to IHI. Across 9,664 patients, 1,598 charts were reviewed. All sites showed improvement with 67% of CHA patients, 92% of Dunn patients, 80% of MHH patients, and 60% of HHS having complete 4Ms assessments by April 2025. Progress in acting on the 4Ms varied: 66% of CHA, 33% of Dunn, 45% of MHH, and 58% of HHS patients had 4Ms-informed clinical actions documented. Barriers included integrating 4Ms into Epic workflows, capturing meaningful “What Matters” data, and adapting the model to inpatient and outpatient contexts. Despite challenges, all sites advanced toward AFHS goals. Findings highlight the need for site-specific strategies, continuous data use, and sustained support. Strategies to support automatic data collection and reporting are needed. Linking the 4Ms to risk stratification and performance incentives may further enhance adoption and scalability.

